# Supplementary material for: Rational Design of Novel Single-Atom Catalysts of Transition-Metal-Doped 2D AlN Monolayer as Highly Effective Electrocatalysts for Nitrogen Reduction Reaction
Source: Molecules. 2024 Dec 6;29(23):5768. doi: 10.3390/molecules29235768 (PMC11643860; doi:10.3390/molecules29235768)
Supplement: Supplementary file 1 [file molecules-29-05768-s001.zip › molecules-3342409-supplementary.pdf]

Supplementary Materials

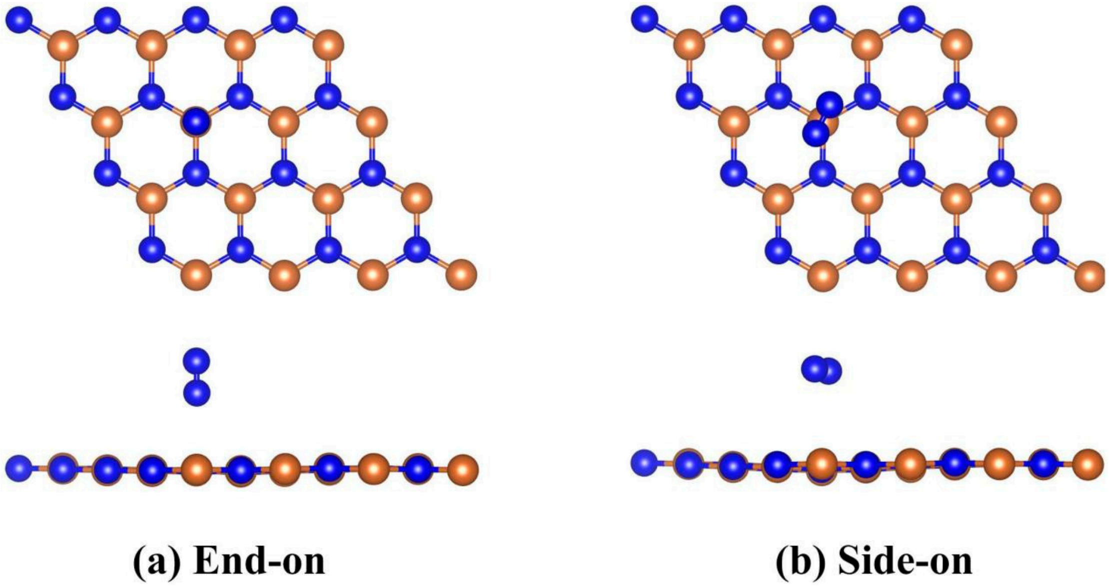

Figure S1. Top and side view of the optimized geometric structures of the adsorption of N_2 on pristine AlN monolayer with the (a) end-on and (b) side-on patterns.

Table S1. Calculated adsorption energies values of N_2 on pristine AlN monolayer with the end-on and side-on patterns .

System	Adsorption energies (eV)	N-N bond lengths (Å)	Al-N distances (Å)
AlN@N_2 end-on	-0.093	1.115	2.727
AlN@N_2 side-on	-0.083	1.115	3.586, 3.537

Table S2.The relative energies (ΔE) of nonmagnetic (NM) and the ferromagnetic (FM) spin polarization, the magnetic moments of FM (M_{tot}) of the different SACs.

	SACs	ΔE (meV)		$M_{tot}(\mu_B)$
		NM	FM	
3d	Sc@AlN	0.0	/	0.0
	Ti@AlN	294.1	0.0	1.0
	V@AlN	1.3	0.0	2.0
	Cr@AlN	0.0	1081.9	3.0
	Mn@AlN	1335.0	0.0	4.0
	Fe@AlN	1523.8	0.0	5.0
	Co@AlN	1069.0	0.0	4.0
	Ni@AlN	573.0	0.0	3.0
	Cu@AlN	391.9	0.0	2.0
	Zn@AlN	90.4	0.0	1.0
4d	Y@AlN	/	/	/
	Zr@AlN	223.4	0.0	1.0
	Nb@AlN	0.0	/	0.0
	Mo@AlN	187.1	0.0	3.0
	Tc@AlN	819.9	0.0	2.0
	Ru@AlN	256.0	0.0	1.0
	Rh@AlN	0.0	/	0.0
	Pd@AlN	157.2	0.0	1.0
	Ag@AlN	254.3	0.0	2.0
	Cd@AlN	113.7	0.0	1.0
5d	Hf@AlN	252.5	0.0	1.0
	Ta@AlN	0.0	/	0.0
	W@AlN	85.6	0.0	0.8
	Re@AlN	940.6	0.0	2.0
	Os@AlN	232.2	0.0	3.0
	Ir@AlN	0.0	/	0.0

	Pt@AlN	129.8	0.0	1.0
	Au@AlN	134.3	0.0	1.0
	Hg@AlN	129.8	0.0	1.0

Table S3. Calculated ΔG_{*H} and ΔG_{*N_2} values of Os@AlN with end-on and side-on adsorption configurations.

System	ΔG_{*H} (eV)	ΔG_{*N_2} (eV)	
		end-on	side-on
Os@AlN	-0.042	-0.074	0.868

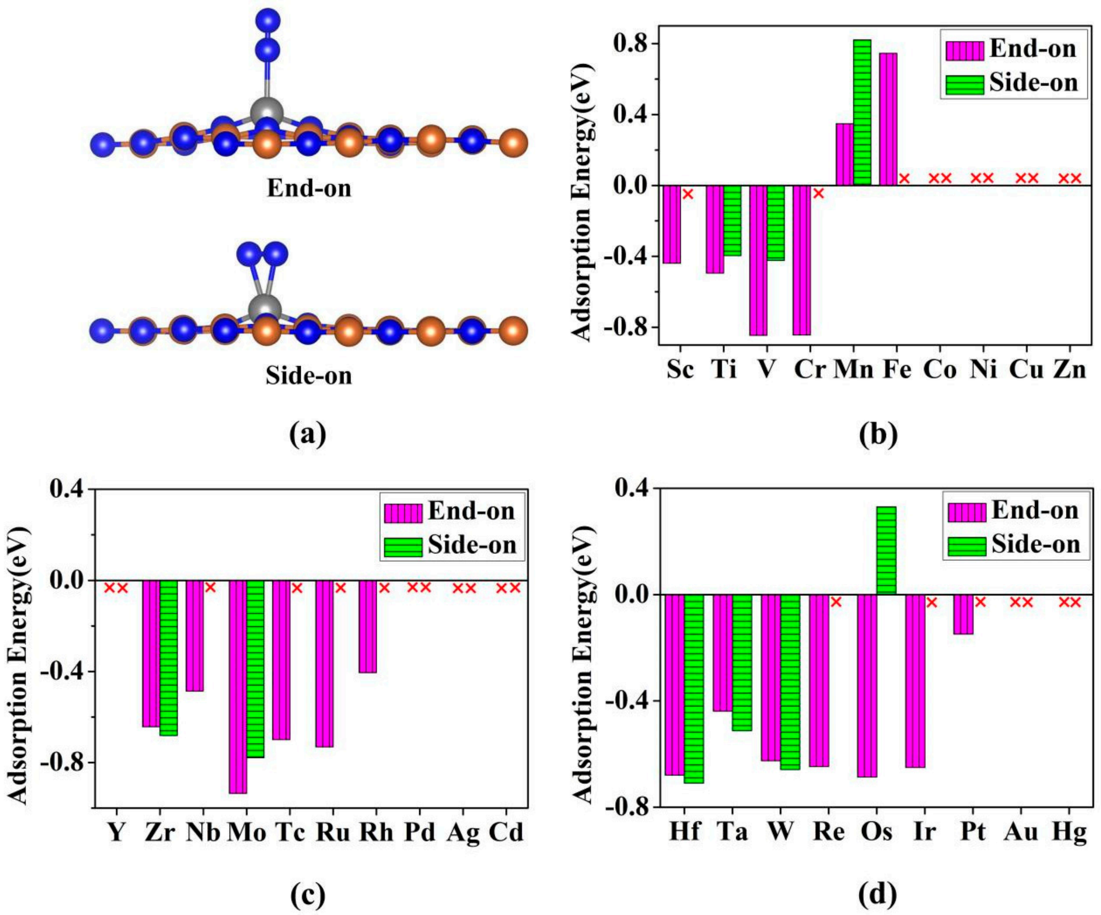

Figure S2. (a) The geometric structures of the adsorption of N_2 on TM@AlN via the end-on and side-on configures. Adsorption energy (E_{ads} in eV) of N_2 adsorbed on (b) 3d TM@AlN, (c) 4d TM@AlN and (d) 5d TM@AlN.

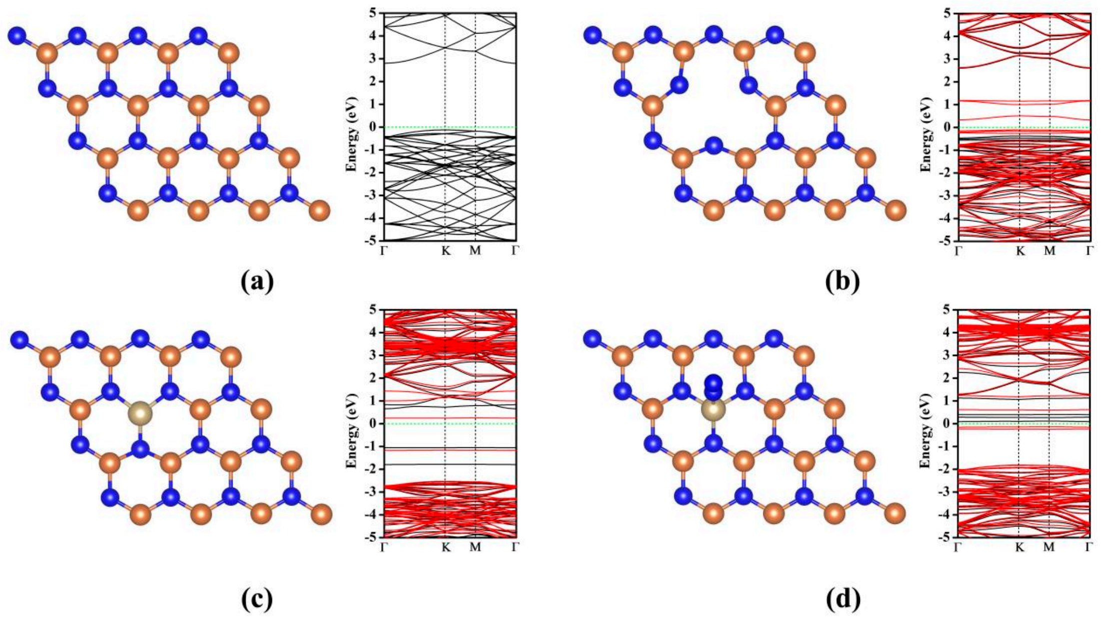

Figure S3. The pristine AlN monolayer (a), defective AlN with Al monovacancy (b), Os@AlN (c) and N_2 adsorption on Os@AlN with end-on configuration (d) of top view and side view, and the corresponding band structures (DFT-PBE level). The black and red dotted lines in the band structures denote the spin-up (↑) and spin-down (↓) channels, respectively. The Fermi-level is set to be zero denoted by green dash line. The orange, blue, and gray denote Al, N, and Os atoms, respectively.

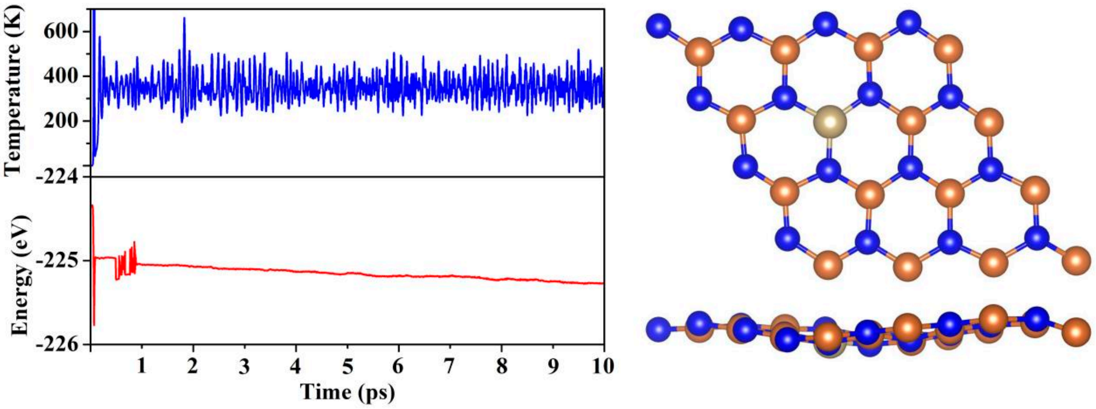

(a) **(b)**

Figure S4. (a) The temperature and energy fluctuations of Os@AlN during 10 ps of AIMD simulation with a time step of 2 fs. (b) The top and side views of Os@AlN after 10 ps of AIMD simulation at T = 350 K.
